# Microsurgical Anatomy of the Anterior Circulation of the Brain Adjusted to the Neurosurgeon’s Daily Practice

**DOI:** 10.3390/brainsci11040519

**Published:** 2021-04-19

**Authors:** Tomas Poblete, Daniel Casanova, Miguel Soto, Alvaro Campero, Jorge Mura

**Affiliations:** 1 Department of Anatomy and Legal Medicine, Medical Faculty, University of Chile, Santiago 8380455, Chile; daniel.casanova@uv.cl (D.C.); momiasoto@gmail.com (M.S.); 2 Department of Neurosurgery, San Borja Arriarán Hospital, Santiago 8360160, Chile; 3 Medical Faculty, University of Valparaiso, San Felipe 2170000, Chile; 4 Department of Neurosurgery, Padilla Hospital, Tucumán T4000, Argentina; alvarocampero@yahoo.com.ar; 5 Department of Neurosurgery, Asenjo Neurosurgical Institute, Santiago 7500691, Chile; jorgemuramd@gmail.com

**Keywords:** cerebrovascular anatomy, anterior circulation, internal carotid artery, cadaveric dissection, pterional approach

## Abstract

The study of cerebrovascular anatomy can be difficult and may take time due to its intrinsic complexity. However, it can also be difficult for the following reasons: the excessive description of neuroanatomy making articles hard to read, the unclear clinical application of what is written, the use of simplified or intricate schematic drawings that are not always appropriate for effective teaching, the poor quality of neuroanatomy dissections and the use of unusual views of figures that are not strictly related to the most frequent neuroimages to be interpreted in daily practice. Because of this, we designed an article that incorporates original and accurate anatomical dissections in an attempt to improve its comprehensibility. Five formalin-fixed adult cadaveric heads, whose vessels were injected with a colored silicone mixture (red for arteries and blue for veins), were dissected and examined under a microscope with magnifications from 3× to 40×. Special emphasis has been placed on correlating topographic anatomy with routine neuroimaging studies from computed tomographic angiography (CTA) and digital subtraction angiography (DSA). The essential surgical anatomy in a neurosurgeon’s daily practice is also described. The cadaveric dissections included in this study contribute to the understanding of the cerebrovascular anatomy necessary for the neurosurgeon’s daily practice.

## 1. Introduction

Cerebrovascular neuroanatomy is very well described in classic neuroanatomy textbooks like those of Rhoton [1], Yasargil [2], Youmans [3], and *Gray’s Anatomy* [4], to name just a few within a large group. Its initial study may prove to be difficult and to take time due to its intrinsic complexity. However, it can also be difficult for the following reasons: the excessive description of neuroanatomy making articles hard to read, the unclear clinical application of what is written, the use of simplified or intricate schematic drawings that are not always appropriate for effective teaching [5], the poor quality of neuroanatomy dissections and the use of unusual views of figures—common practice is to show the circle of Willis from below—that are not strictly related to the most frequent neuroimages to be interpreted in daily practice.

Considering that neurosurgical trainees require a thorough knowledge of neuroanatomy from the very early stages of training to achieve the ability to make accurate interpretations of neuroimages and to translate this information into surgical practice, we designed a clinically focused description of the anterior circulation of the brain that meets the requirements of a neurosurgeon’s daily practice. We incorporated original and accurate anatomical dissections to further improve the recognition of anatomical structures on routine neuroimaging studies from computed tomographic angiography (CTA) and digital subtraction angiography (DSA). The pterional approach with the essential surgical anatomy is also described. Specific anatomic structures that are of special importance in certain clinical scenarios are highlighted. An exhaustive anatomic description of the normal vessels and anatomic variations is beyond the scope of this text and the authors suggest consulting the classic anatomy textbooks for this purpose [1,2,3,4].

## 2. Materials and Methods

### 2.1. Preparation of Specimens

Details of this process have been well-described previously [6,7], so only important steps are mentioned here.

### 2.2. Procurement and Preservation of Cadaveric Head

Human cadavers are obtained from a body donation program associated with the medical faculty of the University of Chile. Once the donor dies, his cadaver is transferred to the Department of Anatomy, where both common carotid arteries and internal jugular veins are dissected and isolated in the neck. Plastic tubes and pipettes of different sizes are then introduced into the lumen of these vessels to later remove the blood and clots with tap water. A preservation solution composed of formalin, phenol and alcohol is then infused, plastic tubes are clamped, and the specimen is stored in a cold room (not freezer). After three weeks the head is cut as low as possible in the neck.

### 2.3. Irrigation

The cadaveric head is translated to the neuroanatomy laboratory, where vertebral arteries are dissected and thin plastic tubes are introduced. Cannulated vessels are flushed with tap water to remove the preservation solution and remaining blood clots until the return fluid is clear of clots and tissue debris. This process usually takes around three days.

### 2.4. Injection

A mixture of silicone, thinner solution, catalyst and color powder paint is prepared. Approximately 100 mL of red mixture is required for the arterial system and 150 mL of blue mixture for the venous system. The colored silicone is injected manually by two people using a single tube silicone-dispensing gun. The specimen is stored in a plastic bag for 48 h to wait for the silicone to cure. After this, the specimen is ready to be dissected.

## 3. Results

In this section we incorporated our cerebrovascular dissections of the anterior circulation of the brain in an attempt to improve its comprehension.

Although there is no consensus regarding the nomenclature of the different segments of the internal carotid artery (ICA), the Bouthillier classification is practical, takes into account clinical and anatomic information and uses a logical numerical scale in the direction of normal blood flow [8]. The supraclinoid portion of the ICA has been divided into three segments according to Gibo, based on the origin of its major branches [9]. Thereby it is possible to describe the eight segments as following.

### 3.1. Cervical

**Figure 1:**
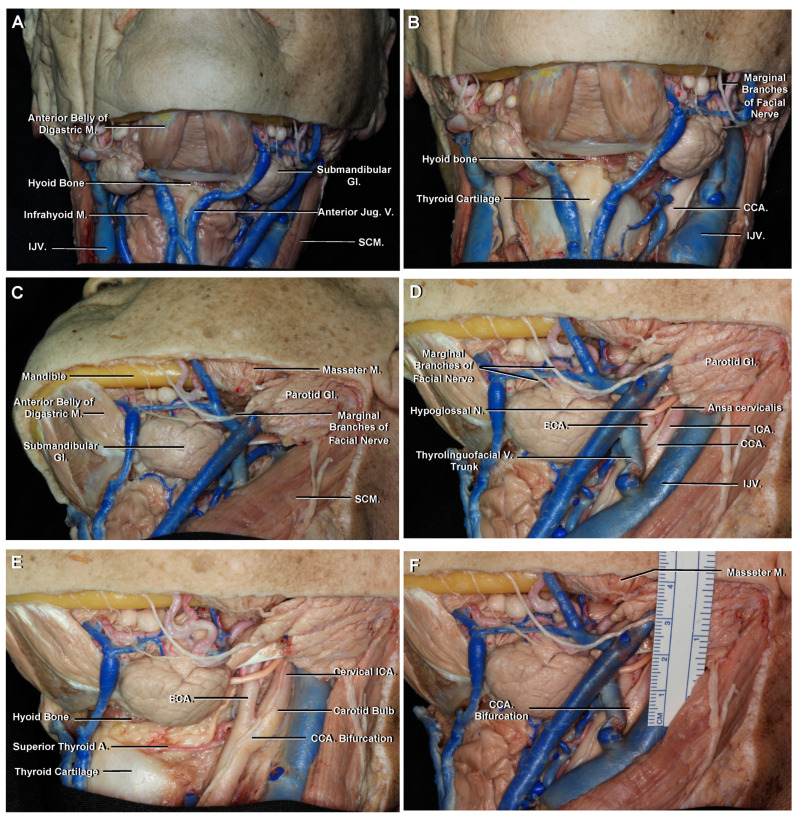
(**A**) Anterior view of the veins of the neck. The anterior jugular veins descend superficially to the infrahyoid muscles. The IJVs are medial to the SCM. (**B**) Enlarged view. Both CCAs run cranially in the carotid space and are medial to the IJV. (**C**) Inferolateral view, left side. The submandibular or digastric triangle is bounded anteriorly by the anterior belly of the digastric muscle, posteriorly by the posterior belly of the digastric muscle and superiorly by the mandible. (**D**) Lateral view, left side. Farabeuf’s triangle is bounded anteriorly by the thyrolinguofacial venous trunk, posteriorly by the IJV and superiorly by the hypoglossal nerve. The CCA bifurcation is seen inside this triangle. (**E**) The left CCA bifurcation is seen approximately at the level of the hyoid bone or superior border of the thyroid cartilage. At its origin, the left cervical ICA lies posterolateral to the ECA and ascends without giving off any branches. (**F**) The mandible angle is covered by the masseter muscle. The CCA bifurcation can be roughly estimated as being two finger-breadths below the mandible angle, approximately 4 cm. Abbreviations: M., muscle; Gl., gland; Jug., jugular; V., vein; IJV., internal jugular vein; SCM., sternocleidomastoid muscle; CCA., common carotid artery; N., nerve; ECA., external carotid artery; ICA., internal carotid artery.

The common carotid artery (CCA) is the largest artery of the neck and originates from the brachiocephalic trunk on the right side and directly from the aortic arch on the left side. Both CCAs run cranially in the carotid space and are located medial to the internal jugular vein (IJV) (Figure 1B). Approximately at the level of the hyoid bone or superior border of the thyroid cartilage, usually between the C3 and C5 vertebral bodies, each CCA bifurcates into the ICA and external carotid artery (ECA) (Figure 1E) [10]. However, the bifurcation can occur as high as C1 or as low as T2 [11].

Farabeuf’s triangle has been mentioned as a good surgical landmark to identify the CCA bifurcation [12]. It is limited posteriorly by the IJV, anteriorly by the thyrolinguofacial venous trunk draining into the IJV and superiorly by the hypoglossal nerve (Figure 1D,F). However, the CCA bifurcation can be located inside or inferior to this triangle [13].

The carotid bulb is the first portion of this segment and has a smooth dilatation at its origin that is known to be involved in blood pressure regulation. The second portion is the cervical ICA. At its origin, the ICA lies posterolateral to the ECA, then it turns medially and ascends towards the carotid canal without giving off any branches (Figure 1E).

### 3.2. Petrous

**Figure 2:**
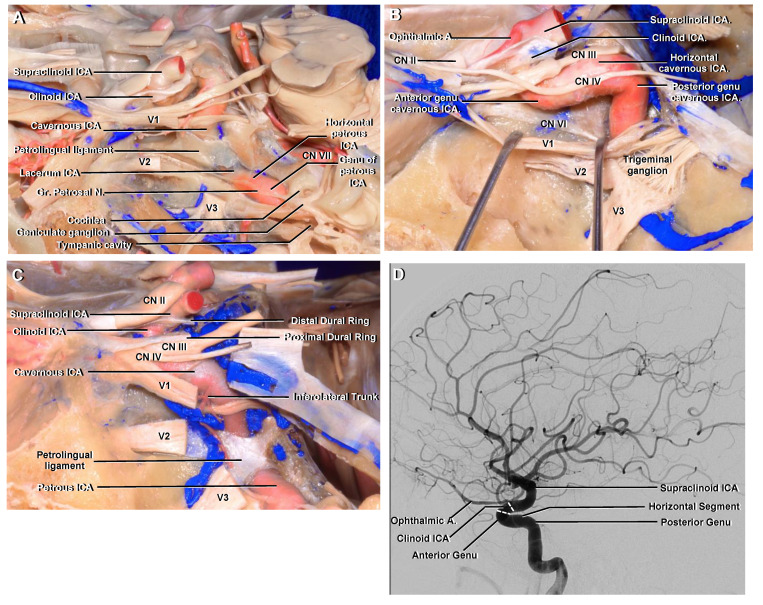
Left middle fossa and cavernous sinus. (**A**) The dura of the middle fossa floor has been removed and part of the trigeminal ganglion and its 3 divisions has been cut. It is possible to appreciate an ossified petrolingual ligament. The petrous ICA is positioned lateral to the trigeminal nerve, and the greater petrosal nerve courses above its horizontal segment. (**B**) Another specimen. The ophthalmic nerve (V1) has been reflected inferiorly. The cavernous ICA is related laterally with the oculomotor, trochlear and ophthalmic nerves. The abducens nerve courses inferolaterally to the horizontal segment. (**C**) The cavernous ICA extends proximally from the upper margin of the petrolingual ligament inferiorly to the proximal dural ring superiorly. The inferolateral trunk arises from its horizontal portion. (**D**) Lateral ICA angiogram to be correlated with (**B**,**C**). It is possibly to identify a posterior genu, a horizontal segment and an anterior genu. Abbreviations: Gr., greater; A., artery; CN., cranial nerve. See Figure 1 for other abbreviations.

This segment begins at the entrance into the carotid canal of the temporal bone. It has a short vertical and a longer horizontal portion. At first it ascends vertically and then the horizontal segment courses anteromedially along the long axis of the petrous temporal bone to its apex. The transition between the vertical and horizontal segment forms a knee or genu that relates to important anatomical structures (Figure 2A). The tympanic cavity lies posterolaterally, the cochlea and geniculate ganglion posterosuperiorly and the greater petrosal nerve superficially [14]. The horizontal portion ends at the posterior edge of the foramen lacerum.

### 3.3. Lacerum

This segment begins where the carotid canal ends, which is at a vertical line at the posterolateral margin of the foramen lacerum [8]. The foramen lacerum is an opening on the exocranial surface of the skull base that, in life, is filled with fibrocartilaginous tissue. The lacerum ICA courses above and does not pass through the foramen lacerum [14]. This segment is inferomedial to the trigeminal nerve and ganglion, above the foramen lacerum and in the lateral wall of the sphenoid sinus (Figure 2A). The artery then turns upward around the medial edge of the petrous apex and ascends in a posterior extension of the carotid sulcus and ends at the upper edge of the petrolingual ligament. This ligament is a continuation of the periosteum of the carotid canal that extends from the lingual process of the sphenoid bone anteriorly to the petrous apex posteriorly.

### 3.4. Cavernous

The cavernous ICA begins at the upper edge of the petrolingual ligament [8]. Its trajectory forms a vertical posterior genu, passes forward in a horizontal direction and ends with a vertical anterior genu in the proximal dural ring (Figure 2A–C). The proximal dural ring is the dura lining the lower surface of the anterior clinoid process and is part of the roof of the cavernous sinus [15]. This segment runs inside the cavernous sinus and relates laterally with the oculomotor, trochlear, ophthalmic and abducens nerves (Figure 2B).

This segment gives origin to two branches: the first is the meningohypophyseal trunk that supplies the neurohypophysis, and the second is the inferolateral trunk that supplies the dura of the inferolateral wall of the cavernous sinus.

### 3.5. Clinoid

**Figure 3:**
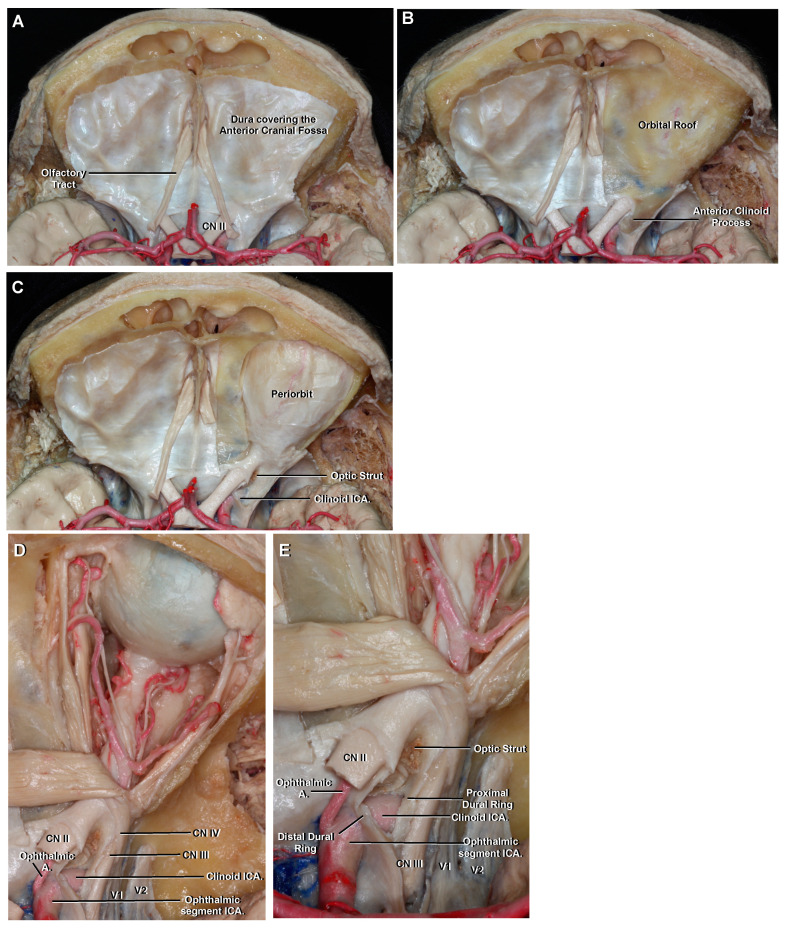
(**A**) Anterior cranial fossa, superior view. The dura is seen covering both orbital roofs. (**B**) The dura covering the right orbital roof has been removed to show the anterior clinoid process. The supraclinoid ICA enters the subarachnoid space medial to the anterior clinoid process and follows a posterior and slightly superior trajectory. (**C**) The roof of the right orbit has been removed to expose the periorbit. The right anterior clinoid process has been removed to expose the clinoid ICA. (**D**) The periorbit and orbital fat has been removed. The ophthalmic artery originates from the superomedial surface of the supraclinoid ICA, below the optic nerve, and then runs anteriorly to enter the optic canal. At the orbital apex, it lies inferolateral to the optic nerve and then continues forward, crossing above it with an anteromedial trajectory. (**E**) Enlarged view. The clinoid ICA is the shortest of all ICA segments and is located between the proximal and distal dural rings. The optic canal and superior orbital fissure have been unroofed and the optic strut is identified separating them.

The clinoid segment is the shortest of all the ICA segments. It comprises only a small, wedge-shaped area along the superior aspect of the anterior genu of the cavernous ICA; it begins at the proximal dural ring and ends in the distal dural one (Figure 2C and Figure 3) [10]. The dura from the anterior portion of the cavernous sinus and tentorium splits into a proximal and distal dural ring that surrounds the inferior and superior surface of the anterior clinoid process, respectively. Thus, this segment is an interdural structure and the only way to expose it is by removing the anterior clinoid process (Figure 3) [16]. This procedure is called anterior clinoidectomy. Both dural rings are not really a natural anatomic structure, but a surgical construct created by removing the anterior clinoid process and doing a circumferential dural incision around the ICA [17].

### 3.6. Ophthalmic

**Figure 4:**
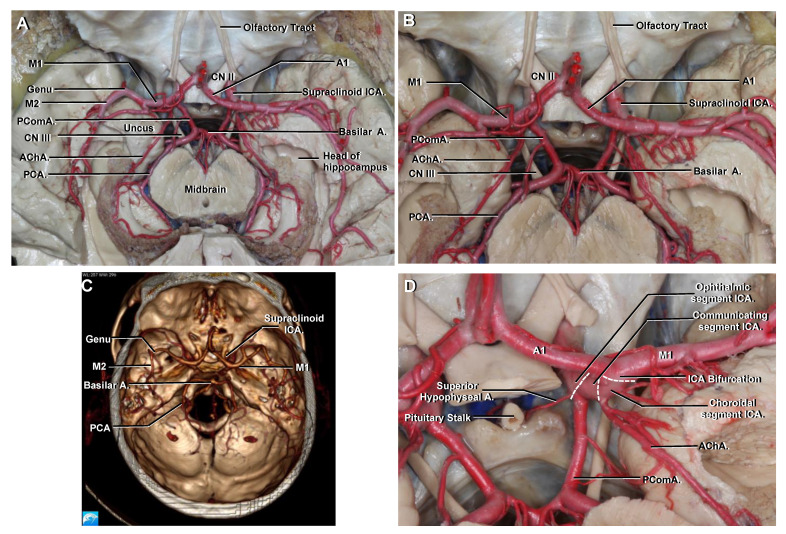
Superior view to demonstrate the supraclinoid ICA, circle of Willis and adjacent structures as seen in an axial CTA. (**A**) The circle of Willis lies above the sella turcica and surrounds the infundibular stalk. The apex of the uncus of the temporal lobe is seen pointing towards the oculomotor nerve. The PComA arises from the posterior surface of the communicating ICA and connects the anterior with the posterior circulation. The AChA also arises from the posterior surface of the ICA and is intimately related to the uncus of the temporal lobe. (**B**) Enlarged view from A. The PComA lies superomedial to the oculomotor nerve and has a roughly parallel trajectory to it. (**C**) Axial CTA VR reconstruction to be correlated with A and B. It is possible to identify the main intracranial arteries but not small branches like the AChA or PComA. (**D**) Enlarged view seen from the left. The three segments of the supraclinoid ICA are seen. The AChA arises a few millimeters distal to the PComA origin. Abbreviations: CTA., computed tomographic angiography; PComA., posterior communicating artery; VR., volume-rendered; AChA., anterior choroidal artery; PCA., posterior cerebral artery. See Figure 1, Figure 2 and Figure 3 for other abbreviations.

The ophthalmic segment is the first of three portions of the supraclinoid ICA. It begins at the distal dural ring where the artery perforates the dura, at the medial aspect of the anterior clinoid process, to enter the subarachnoid space and then follow a posterior and slightly superior trajectory to just proximal to the origin of the posterior communicating artery (PComA) (Figure 3D,E) and (Figure 4) [1].

This segment gives origin to two important arterial branches: the ophthalmic and superior hypophyseal arteries.

The ophthalmic artery arises medial to the anterior clinoid process. It originates from the superomedial surface of the ICA, below the optic nerve, and then runs anteriorly to enter the optic canal [16]. At the orbital apex the artery lies inferolateral to the optic nerve and then continues forward, crossing over it with an anteromedial trajectory (Figure 3D,E). In approximately 8% of cases, the ophthalmic artery may originate from the clinoid or cavernous segment [1].

The superior hypophyseal arteries are a group of an average of two small perforating arteries that arise from the posterior or medial aspect of the artery (Figure 4D) [1]. These arteries are most commonly distributed to the pituitary stalk, anterior pituitary lobule and, less commonly, to the optic nerves, chiasm and floor of the third ventricle. The largest of the branches is often referred to as the superior hypophyseal artery.

### 3.7. Communicating

The communicating segment is the second of the three portions of the supraclinoid ICA. It extends from the origin of the PComA to just proximal to the origin of the anterior choroidal artery (AChA) (Figure 4) [1,9].

**Figure 5:**
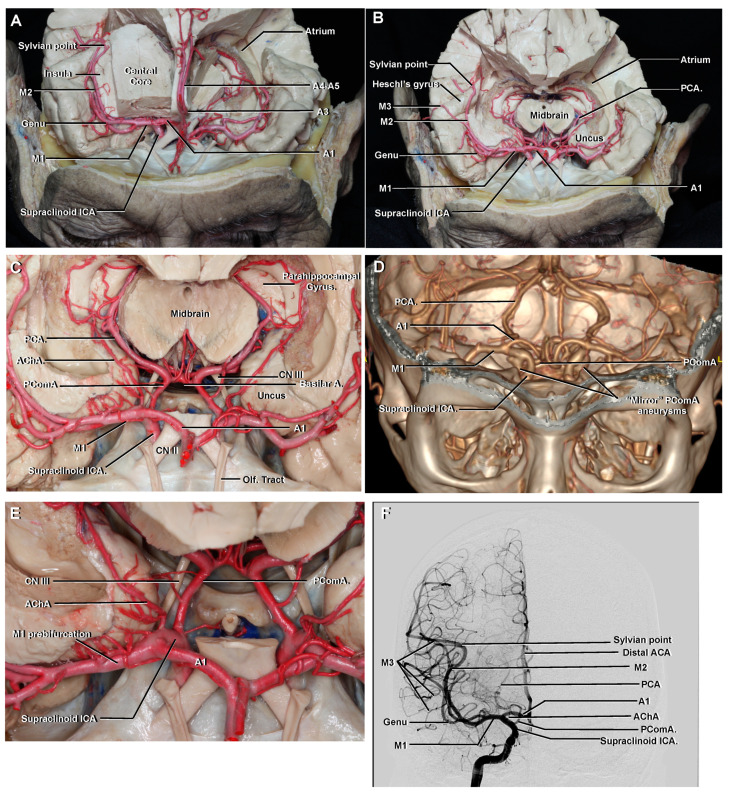
(**A**) Anteroposterior view. The supraclinoid ICA and its terminal branches are clearly seen. M1 originates at the ICA terminal bifurcation, courses with a horizontal trajectory and ends at the genu. M2 begins at the genu and courses with an anterior to posterior trajectory along the insular cortex. A1 courses with a horizontal trajectory to join the AComA. (**B**) The central core has been removed to demonstrate the arterial structures related to the mesial temporal lobe. (**C**) Enlarged view. The uncus and parahippocampal gyrus are limited medially by the PCA. (**D**) Coronal CTA VR reconstruction to be correlated with (**A**,**C**). Bilateral PComA aneurysms (“mirror aneurysms”) are seen arising immediately distal to the origin of the PComA, and they project posterolaterally towards the oculomotor nerve. (**E**) Enlarged view from (**C**). The AChA originates from the posterior surface of the ICA distal to the PComA, but proximal to the ICA bifurcation, and is closely related to the uncus. (**F**) Anteroposterior view of a right ICA angiogram to be correlated with (**A**,**C**). The central core of the cerebral hemisphere is located in the rectangle limited anteriorly by the A1, M1 and genu of the MCA, medially by the distal anterior cerebral artery (A2–A5 segments), laterally by the M2 segment and posteriorly by a transverse line drawn from the sylvian point.

The PComA arises from the posterior surface of the ICA. It runs posteriorly and medially, below the floor of the third ventricle, to join the posterior cerebral artery (PCA) in a close relationship with the oculomotor nerve, therefore connecting the anterior (carotid) with the posterior (vertebrobasilar) circulation (Figure 4 and Figure 5). If the configuration of the PComA is small or normal size, it courses superomedially to the oculomotor nerve. However, if the PComA is the main supply of the PCA, a configuration, called fetal PCA, that occurs in as many as 20% patients, it courses further laterally above or lateral to the oculomotor nerve [1].

The PComA gives origin to an average of eight perforating branches, mostly from the superior surface that course superiorly to enter the floor of the third ventricle.

### 3.8. Choroidal

The choroidal segment is the last of the three segments of the supraclinoid ICA. It extends from the origin of the AChA to the ICA bifurcation (Figure 4D) [1,9].

The AChA arises from the posterior surface of the ICA closer to the origin of the PComA than to the ICA bifurcation. It is usually a single artery rarely larger than 1 mm in diameter [18]. It arises lateral to the optic tract and then courses posteromedially in the carotid cistern to reach the optic tract from below [19]. From this point the AChA follows the general direction of the optic tract between the mesial temporal lobe and the cerebral peduncle (Figure 4 and Figure 5) [2]. Then it turns laterally and passes through the choroidal fissure to enter the choroid plexus of the temporal horn [1,10,20].

The perforating branches of the AChA deserve special considerations because of the critical neural structures that they supply. These branches supply, in decreasing order of frequency, the optic tract, uncus, middle third of the cerebral peduncle, hippocampus, lateral part of the geniculate body, inferior half of the posterior limb of the internal capsule and most of the globus pallidus [1,19,21].

### 3.9. Terminal Branches of the ICA

#### 3.9.1. Middle Cerebral Artery (MCA)

The MCA is the largest of the two terminal branches of the ICA. It is divided into four segments (Figure 4 and Figure 5) [1,22,23]:

#### 3.9.2. M1

The M1 segment arises at the ICA bifurcation, below the anterior perforated substance, and runs laterally with a horizontal course in the sylvian fissure towards the insula. It is also called the sphenoidal segment, because it runs parallel to and approximately 1 cm posterior to the sphenoid ridge. This segment ends at the genu, a 90-degree turn in the artery as it courses over the limen insulae, where the artery changes from a horizontal to an anterior to posterior axis [22].

The main trunk of the M1 segment bifurcates distally into superior and inferior trunks in 78% of cases, trifurcates into superior, middle and inferior trunks in 12% of cases and divides into multiple trunks in 10% of cases [1]. Thus, the M1 segment is subdivided into a prebifurcation and postbifurcation part. An average of 10 perforating branches arise from the superior surface of the M1 prebifurcation and are called lateral lenticulostriate perforators. The earlier the bifurcation, the greater is the number of postbifurcation perforators [1]. These perforators supply the superior half of the anterior and posterior limbs of the internal capsule, body and head of the caudate nucleus, putamen and the lateral part of the globus pallidus [21]. The small cortical branches arising from the M1 prebifurcation are referred to as early branches. Nearly 50% of MCAs send early branches to the temporal lobe, but less than 10% give early branches to the frontal lobe [23].

#### 3.9.3. M2

Also called the insular segment, the M2 segment begins at the genu and runs over the surface of the insula in the circular sulcus, also called the limiting sulcus, with an anterior to posterior axis, then ends where the arteries make their next 90-degree turn. Perforators of the M2 segment supply the insular cortex.

#### 3.9.4. M3

Also called the opercular segment, the M3 segment begins at the genu where the arteries leave the insular surface, then run over the surface of the frontoparietal and temporal opercula to reach the cortical surface of the sylvian fissure.

#### 3.9.5. M4

Also called the cortical segment, the M4 segment begins as soon as the arteries reach the cortical surface of the sylvian fissure, to their final territory. These branches supply the lateral two-thirds of the lateral surface of the hemisphere.

### 3.10. Anterior Cerebral Artery (ACA)

**Figure 6:**
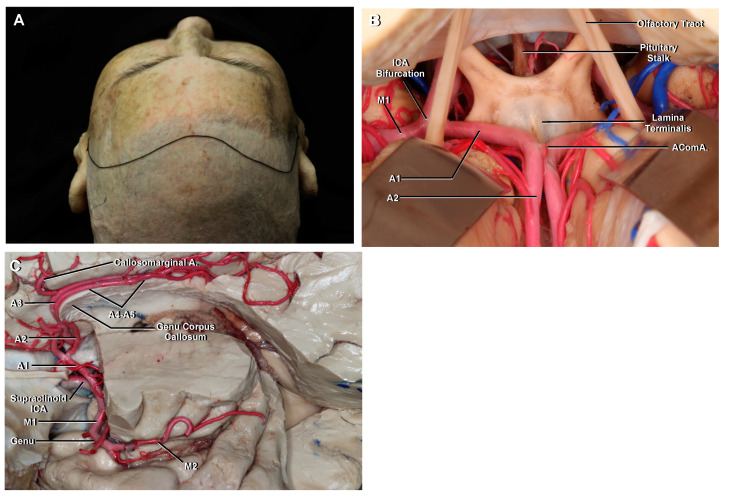
(**A**) Cerebrovascular anatomy as seen with a bicoronal and an interhemispheric approach to the region of the AComA. (**B**) The A1 originates from the ICA bifurcation and runs medially with a horizontal course above the optic chiasm, to join the AComA in the interhemispheric fissure. The A2 segment extends vertically from the AComA and follows the rostrum of the corpus callosum. (**C**) Another specimen. Superolateral view, left side. The different segments of the ACA are possible to identify, from the ICA bifurcation to its distal course in the pericallosal sulcus. The pericallosal artery is referred to as the ACA segment distal to the AComA that courses in the pericallosal sulcus. The callosomarginal artery courses in the cingulate sulcus.

The ACA is the smallest of the two terminal branches of the ICA. It is divided into five segments (Figure 6) [1]:

#### 3.10.1. A1

Also called the precommunicating segment, the A1 segment arises at the ICA bifurcation, below the anterior perforated substance, and runs medially, with a horizontal course above the optic nerve or chiasm, to join the anterior communicating artery (AComA) in the interhemispheric fissure. An average of eight perforating arteries arise from the A1 segment; these are called medial lenticulostriate arteries, and they supply the region of the anterior hypothalamus and medial third of the anterior commissure [1].

The recurrent artery of Heubner is the largest and longest ACA perforating branch. It arises from the distal A1 or proximal A2. In its course, the artery of Heubner runs back parallel to A1 and passes above the ICA bifurcation to enter the anterior perforated substance. It supplies the inferior half of the anterior limb of the internal capsule and adjacent portions of the caudate nucleus, putamen and globus pallidus [21].

The AComA also gives rise to one or two perforating arteries that mainly supply the anterior wall of the third ventricle and hypothalamic area [24].

#### 3.10.2. A2

Also called the postcommunicating or infracallosal segment, this segment extends vertically from the AComA, passes anterior to the lamina terminalis and ends at the junction of the rostrum and genu of the corpus callosum.

#### 3.10.3. A3

Also called the precallosal segment, the A3 segment curves around the genu of the corpus callosum.

#### 3.10.4. A4 and A5

The A4 and A5 segments are the supracallosal and postcallosal segments, respectively, and are divided in a vertical plane by the coronal suture. They course above the corpus callosum, under the free margin of the falx cerebri.

The callosomarginal artery is the largest branch of the ACA. It courses in the cingulate sulcus. Its most frequent origin is from A3, but it may also arise from A2 or A4.

The pericallosal artery is referred to as the ACA segment distal to the AComA that courses in the pericallosal sulcus.

The ACA typically supplies the medial third of the lateral surface of the hemisphere and anterior two-thirds of the medial brain surface.

### 3.11. Circle of Willis

The circle of Willis is the principal anastomotic arterial ring at the base of the brain between the anterior and posterior circulation [4]. It lies above the sella and pituitary gland and surrounds the infundibular stalk and mammillary bodies. When the circle of Willis is complete, it has 10 components and is comprised sequentially in an anterior to posterior direction by: the AComA, both A1, both ICA, both PComA, both P1 and the basilar artery. Anatomic variations are the rule and the PComA is the most common site of these variations [1].

## 4. Discussion

### 4.1. In Relation to Different ICA Classifications

The first classification of the ICA was published by Fischer in 1938 and was based on the angiographic course of the intracranial ICA, rather than its arterial branches [25]. It described five segments in the opposite direction of blood flow. Gibo in 1981 proposed an anterograde classification of the ICA that divided the ICA into cervical, petrous, cavernous and supraclinoid segments. The supraclinoid ICA was further divided into ophthalmic, communicating and choroidal parts [9]. In 1996 Bouthillier proposed a classification that incorporated adjacent anatomic structures and clinical considerations, numbering different segments in the direction of anterograde blood flow [8]. According to this classification they identified seven segments: cervical (C1), petrous (C2), lacerum (C3), cavernous (C4), clinoid (C5), ophthalmic (C6) and communicating (C7). New classification systems have been proposed incorporating ventral endoscopic observations and new anatomical landmarks [26].

Although there is no consensus regarding the classification of the ICA, we used the classification proposed by Bouthillier [8], because it is commonly used at present, and described the supraclinoid ICA according to Gibo [9]. Neither classification is mutually exclusive; conversely, they can complement each other, providing a more descriptive and logical classification of the ICA. Because the AChA supplies critical neural structures, we considered it important to highlight.

Special consideration is deserved by the clinoid segment of the ICA, because it represents the transition segment between the extradural and intradural ICA. Distinguishing aneurysms of the cavernous segment (extradural) from those that involve the supraclinoid (intradural) ICA is of importance for understanding clinical presentation and treatment options [27]. Ruptured aneurysms of the supraclinoid ICA typically present as subarachnoid hemorrhages and may be treated by surgical or endovascular occlusion. Conversely, aneurysms of the cavernous ICA pose little or no risk of subarachnoid hemorrhage, but, when ruptured, can lead to the development of a high-flow carotid-cavernous fistula, whose treatment is generally endovascular when there is a risk of visual impairment.

The ophthalmic artery has traditionally served as a landmark in distinguishing extradural from intradural aneurysms [10]. However, as mentioned before, its origin is variable and may have a more proximal origin in up to 8% cases [1]. Because of this, Gonzalez proposed the inferior border of the optic strut as identified on CTA as a more reliable anatomic location to discriminate between intradural and extradural aneurysms [27].

### 4.2. Anatomic Information as Applied to Angiography

#### Anteroposterior Projection (AP)

Digital subtraction angiography (DSA) remains the gold-standard diagnostic modality for intracranial vascular lesions [28]. Properties such as the possibility of obtaining precise 3-dimensional (3D) images of vascular lesions, their blood supply and venous drainage are unique advantages of this imaging modality.

Depending on its development, the PComA can be injected only in the ICA angiogram, in the vertebrobasilar angiogram or in both. To better understand the topographic anatomy on an angiogram, it is useful to consider a hypoplasic or fetal type of the PCA (Figure 5F). In the AP projection of an ICA angiogram, the PComA is seen arising behind the supraclinoid ICA, and it courses medially and posteriorly to join the PCA (Figure 5C,F). Similarly, the initial segment of the AChA is seen arising from the medial or posteromedial aspect of the supraclinoid ICA, distal to the PComA origin but proximal to the ICA bifurcation. It runs first medially, curves around the uncus of the temporal lobe and then turns laterally to enter the temporal horn.

The ICA bifurcation and the M1 segment are always easily recognizable on an AP angiogram. In the usual configuration, the M1 segment follows a relatively straight course to the genu. The M2 segment is seen coursing upward over the insular surface, and it ends where the arteries make their next 90-degree turn running over the frontal, parietal and temporal opercula. The highest and most medial M3 branch that leaves the insula and turns laterally is termed the angiographic sylvian point (Figure 5A,B) [22].

When anatomic dissections are correlated with an angiogram, it is possible to identify the insula medial to M2 branches and the frontal, parietal and temporal opercula lateral to them (Figure 5A). The central core of the cerebral hemisphere (composed of the insular surface, basal ganglia, thalamus, internal, external and extreme capsules and claustrum) is located in the rectangle limited anteriorly by the A1, M1 and genu of the MCA, medially by the distal anterior cerebral artery (A2–A5 segments), laterally by the M2 segment and posteriorly by a transverse line drawn from the sylvian point (Figure 5A,F) [29]. The sylvian point is always located above the medial end of Heschl’s gyrus and always points toward the atrium of the lateral ventricle. In relation to the uncus of the temporal lobe, the anterior segment is projected behind the M1 segment, the apex points to the oculomotor nerve and supraclinoid ICA, and the posterior segment and parahippocampal gyrus of the temporal lobe lie lateral to the PCA (Figure 5C) [20].

### 4.3. Anatomic Information as Applied to Computed Tomographic Angiography (CTA)

Neurosurgeons are frequently faced with the need to interpret brain CTA, especially in emergency settings when angiography is limited. CTA has proven to be useful for the detection of intracranial aneurysms larger than 3 mm [30]. Because of its less invasive neurovascular imaging modality, availability, cost-effectiveness and ability to be performed quickly, it has become the first-line choice for subarachnoid hemorrhage in many institutions.

The vascular arterial anatomy can be addressed with a CTA reformatted into axial, sagittal and coronal maximal intensity projection (MIP) imaging and volume-rendered (VR) three-dimensional reconstructions. In the axial CTA VR reconstruction, it is possible to identify the whole ICA trajectory from its origin at the common carotid bifurcation up to its terminal bifurcation, ACA, AComA complex and MCA up to its M2 segment.

A major benefit of CTA over DSA is to relate vascular anatomy to the bone structures of the skull base. Of particular importance is the proximity of the anterior clinoid process to a given aneurysm. Most aneurysms arising from the clinoid and ophthalmic segment, but also some specific aneurysms originating from the communicating segment close to the tip of the anterior clinoid process require partial or total removal of the anterior clinoid process to expose the clinoid ICA and get proximal control or to expose the neck of the aneurysm [31]. This preoperative information is only possible to predict with a detailed analysis of the CTA and its adjacent bone structures.

One of the most frequent types of cerebral aneurysms are PComA aneurysms. These aneurysms arise from the posterior wall of the ICA, immediately distal to the origin of the PComA, and usually project posterolaterally (Figure 5D). Their close relationship with the oculomotor nerve, as can be seen by correlating Figure 5D with Figure 5C, explains why these aneurysms may compress the oculomotor nerve.

The most important limitation of CTA is the inability to visualize very small vessels, including the ophthalmic, superior hypophyseal, AChA and perforators. Li Lu et al. reported inferior spatial resolutions of CTA (0.6 mm), compared to those of DSA (0.32 mm) [30]. Neither is it possible to obtain information regarding flow rates or collateral circulation using standard CTA sequences.

### 4.4. Anatomic Information as Applied to Surgery 

One of the most important approaches in neurosurgery is the pterional approach, which was popularized by Yasargil in the 1970s; it is widely used to expose aneurysms of the anterior circulation and other vascular and nonvascular lesions [32].

**Figure 7:**
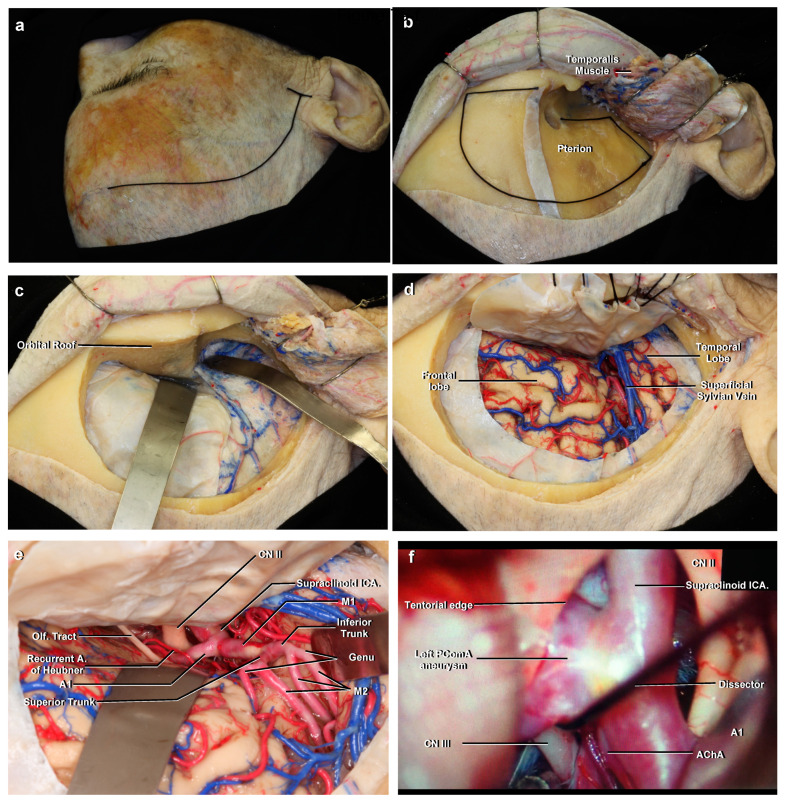
(**a**) The positioning and the skin incision for the classic pterional approach. Right side. (**b**) After the scalp flap is made to protect the temporal branches of the facial nerve, the temporalis muscle is reflected posteroinferiorly to expose the pterion. A frontotemporosphenoidal craniotomy is planned. (**c**) After the removal of the bone flap, the orbital roof and lesser wing of the sphenoid bone are drilled. (**d**) After opening the dura, the frontal and temporal lobes and superficial sylvian veins are exposed. (**e**) After the sylvian fissure has been widely opened, the main neurovascular structures are seen. (**f**) Left pterional approach during treatment of the left PComA aneurysm previously shown in Figure 5D. A dissector is gently pressuring upward to demonstrate the proximity of the distal neck to the origin of the AChA.

The pterional approach begins with a curvilinear incision made immediately anterior to the tragus, just behind the hairline, and continues with one of several different types of flaps designed to preserve the temporal branches of the facial nerve and expose the pterion (Figure 7) [33,34]. Then a frontotemporosphenoidal craniotomy is performed, and the sphenoid wing and orbital roof are drilled with the objective of obtaining a flat surface over the orbit connecting the anterior and middle cranial fossa. The dura is opened with a semicircular incision and then the approach proceeds with opening the sylvian fissure and basal cisterns. As can be seen from a superolateral view with a right pterional approach and a wide opening of the sylvian fissure (Figure 7E), it is possibly to identify the supraclinoid ICA, ICA bifurcation, M1, A1, superior and inferior trunks of M1, genu of the MCA, M2 segment and the recurrent artery of Heubner.

The left pterional approach shown in Figure 7F corresponds to an intraoperative image during treatment of the left PComA aneurysm of Figure 5D. Clipping of these aneurysms may be considered straightforward because of their simple anatomy. However, they may be associated with significant morbidity. Unnoticed clipping of the AChA resembles a total M1 occlusion because of the perforating vascular territory it supplies. The main difference is the absence of cortical involvement. 

Occasionally it may also be necessary to expose the cervical ICA. It is frequently prepared or exposed prior to a craniotomy for the treatment of aneurysms originating from the clinoid or ophthalmic segment of the ICA to ensure proximal vascular control [35]. The cervical ICA may be exposed with a horizontal incision centered two finger-breadths below the mandible angle, approximately 4 cm, just anterior to the anterior margin of the sternocleidomastoid muscle (Figure 1F). Once the bifurcation of the CCA has been exposed, the ICA is identified, posterolateral to the ECA. A preoperative cervical CTA may be useful to rule out higher or lower bifurcations.

## 5. Conclusions

The cadaveric dissections included in this study demonstrate the main topographic and neurovascular relationships necessary to assess routine neuroimaging tests correctly and introduce surgical anatomy with important clinical pearls. They contribute to the understanding of the cerebrovascular anatomy necessary for the neurosurgeon’s daily practice.

## Data Availability

Data are contained within the article.

## Abbreviations

CCA., common carotid artery; ECA., external carotid artery; ICA., internal carotid artery; PComA., posterior communicating artery; AChA., anterior choroidal artery; MCA., middle cerebral artery; ACA., anterior cerebral artery; AComA., anterior communicating artery; CTA., computed tomographic angiography; DSA., digital subtraction angiography; IJV., internal jugular vein; PCA., posterior cerebral artery; MIP., maximal intensity projection; VR., volume-rendered.
